# Progress in Bacteriology

**Published:** 1902-11-01

**Authors:** 


### Progress in Bacteriology.

**Typhoid Bacilli and the Soil.**—Martin[1] has made a series of experiments to ascertain whether the typhoid bacillus is capable of prolonged life in ordinary soils under natural conditions. Although the B. typhosus does not spore, it has considerable resisting power, a very rapid rate of reproduction, and means of locomotion. In sandy, peaty, and uncultivated soils the bacillus dies rapidly, but in cultivated soils it multiplies and spreads quickly, and will resist high desiccation. This, no doubt, is due to the nitrogenous matter in the cultivated soil, which provides a patulum for the organism. An excess of water in the soil causes a disappearance of the B. typhosus, probably owing to the increase of other bacterial forms whose products are inimical to its growth. All experiments show that the life of the typhoid bacillus in the soil can only be a brief one. The conclusions of Forth and Horrocks[2] differ somewhat from the above. They state that the typhoid bacillus does not spread when placed in the soil, though it may be washed through layers of as much as 18 inches in thickness. Whilst agreeing with Martin that it is more difficult to recover the bacillus from a damp than from a dry soil, they do not find that the presence of organic matter plays any part in determining its existence, but consider the permeability a factor of more importance. Their experiments show that the typhoid bacillus can be disseminated by dust, and that it survives prolonged exposure to direct sunlight. House-flies convey infective matter on their mandibles, legs, wings, and bodies. The results obtained by Pfuhl[3] coincide very closely with the above. He recovered the enteric bacillus from moist garden earth after 88 days, from dry sand after 28 days, and from moist peat after 21 days. Pfuhl isolated the organisms by the Drigalski-Conradi medium. Firth used alkaline litmus-glucose-agar. The question of finding a suitable medium for differentiating the typhoid bacillus, especially for distinguishing between this organism and the B. coli, has long been a difficult one. Drigalski and Conradi used lactose agar to which both neutral red and krystall-violet had been added. In this medium the colonies of B. coli appear red and those of B. typhosus as blue or purple. Grünbaum and Hume[4] have used MacConkey's medium (taurocholate-lactose-agar), and have added to this neutral red (toluylen-red). The taurocholate inhibits the growth of nearly all kinds of intestinal bacteria, whilst in the neutral-red medium the colonies of B. coli appear red and those of B. typhosus as white with a surrounding tint of orange or amber. Makgill[5] states that the B. tetani, B. œdematis maligni and B. mesentericus react on neutral red in the same way. Moore[6] believes that Elsner's plates are the best means of distinguishing between the two organisms B. coli and B. typhosus. Instead of using gelatine, however, he recommends agar, which can be incubated and a quicker result obtained. The foundation of the medium is potato jelly, which, after being made alkaline, has added to it 1 per cent. of potassium iodide. After 24 hours' incubation the typhoid colonies are clear, transparent, and almost invisible with irregular clean-cut margins, the colonies of B. coli opaque, larger and rounded.

**Diphtheria.**—Williams[7] reviews the work of the two bodies of workers who hold diametrically opposite views as to the relationship of true diphtheria bacilli and avirulent atypical forms. The one class believes that diphtheria-like bacilli are simple transitory variations of the species bacillus diphtheriæ varying temporarily in form, cultural characteristics, and virulence, but capable of readily assuming the properties of another form. The other class maintains that certain forms possess such stable properties that one is not converted into the other, and in fact are distinct species. Williams finds that many non-virulent diphtheria-like organisms are stable in their characteristics, and, from a public health point of view, harmless, and that these and the pseudo-diphtheria bacillus are distinct species from the morphologically typical diphtheria bacillus. The typical bacillus may show modifications, which, however, are transitory, and the tendency is to return to the original characteristics. The appearance of non-virulent forms towards the end of an attack of diphtheria may be accounted for by the fact that the true bacillus is dying out and the atypical forms are then noticed. If cultural characteristics are noted and pure cultures made the writer maintains that no variations in species or variety will be found from the beginning to the end of the illness. If normal throats be examined many varieties of diphtheria-like bacilli will be found, but these maintain their cultural characteristics and remain non-virulent. If virulent typical bacilli be found in a normal throat they maintain their characteristics, never changing into pseudo-forms. In an epidemic the character of a bacillus does not undergo modification, there is no gradation into pseudo types, the same type being found in the first case as in the last. Williams failed to modify the characteristics of any species by cultural methods or by transmission through animals or birds. Some very valuable information with regard to the bacteriology of diphtheria is contributed by Woodhead,[8] who has tabulated the results of 27,128 cultures. Of 12,172 cases sent into hospital 73 per cent. gave positive and 26 negative results. In fatal cases where the cultures are taken after death, it is frequently impossible to detect Klebs Löffler bacilli, as they become crowded ont by saprophytic organisms. In laryngeal cases too the difficulty of finding the bacillus is great and generally a single negative result should not be relied upon. The difference in the mortality of cases varies with the type of organism present. Thus with long bacilli the death-rate is 21·4; mixed long and short forms, 20·1; long, short, and irregular forms, 18·6; long and irregular forms, 14·6; irregular forms alone, 17·3; short forms alone, 3·3 per cent. That a fatal faucial angina may result from an infection other than that of the B. diptheria is evidenced by the fact that the death-rate on 565 cases in which streptococci only were found was 5·1; with staphylococci only 13·1; mixed strepto and staphylococci, 10·6 per cent.; and other organisms, 23·7 per cent. Contrary to the findings of previous observers, Woodhead's figures show that in mixed infections the staphylococcus is more dangerous than the streptococcus. The mortality rate when the long diphtheria bacillus was accompanied by the staphylococcus was 32·5 per cent., with the short bacillus and staphylococci 11 per cent. With long bacilli and streptococci the rate was 24·7 per cent., and with short bacilli and streptococci 10·4 per cent. In scarlatinal anginas, too, the staphylococcus infection was the most fatal. With regard to the question of isolation, Dr. Woodhead's figures go to show that diphtheria bacilli may be found in the throat in the majority of cases until the expiration of the eighth week.

[1] Report of Med. Officer of L.G.B., 1900-1. [2] Brit. Med. Jour., Sept. 27, 1902. [3] Zeit. f. Hygien Bd. 40, Hft. 3. [4] Brit. Med. Jour. June 14, 1902. [5] Jour. of Hygiene. October 1901. [6] Brit. Med. Jour., March 27, 1902. [7] Jour. Med. Research, August 1902. [8] Brit. Med. Jour., Sept. 27, 1902.

